# Levels of circulating insulin cell-free DNA in women with polycystic ovary syndrome – a longitudinal cohort study

**DOI:** 10.1186/s12958-019-0478-7

**Published:** 2019-04-05

**Authors:** Pernille Bækgaard Udesen, Anja Elaine Sørensen, Mugdha V. Joglekar, Anandwardhan A. Hardikar, Marie Louise Muff Wissing, Anne-Lis Mikkelsen Englund, Louise Torp Dalgaard

**Affiliations:** 1 Fertility Clinic, Dept. of Gynecology and Obstetrics, Zealand University Hospital, Lykkebækvej 14, 4600 Køge, Denmark; 2 Department of Natural Science and Environment, Universitetsvej 1, 4000 Roskilde, Denmark; 3 Diabetes and Islet Biology Group, NHMRC Clinical Trials Centre, University of Sydney, 92 Parramatta Road, Sydney, NSW 2050 Australia

**Keywords:** Circulating free DNA, Insulin promoter CpG methylation, Demethylation, PCOS, Glucose tolerance, Androgens, Testosterone

## Abstract

**Background:**

Women with Polycystic Ovary Syndrome (PCOS) present a heterogeneous reproductive and metabolic profile with an increased lifetime risk of Type 2 Diabetes (T2D). Early biomarkers of these metabolic disturbances in PCOS women have not been identified. The abundance of circulating insulin gene promotor cell-free DNA (INS cfDNA) was shown to be valuable as a predictive biomarker of β-cell death in individuals with Type 1 diabetes (T1D) as well as with gestational diabetes. Since β-cell death is common to the development of T1D as well as in T2D, we aimed to investigate if insulin-coding DNA is more abundant in circulation of PCOS women (vs Controls) and if their levels change after 6 yr. follow-up as a potential measure to predict future T2D.

**Methods:**

A cohort of 40 women diagnosed with PCOS according to Rotterdam 2003 criteria and eight healthy controls were examined at baseline and 6 years follow-up. Clinical measurements for evaluation of glucose homeostasis as well as blood/serum samples were obtained at each visit. Methylated and unmethylated INS cfDNA were quantified using droplet digital PCR. Differences between groups were assessed using Kruskall-Wallis test and Wilcoxon Signed rank test.

**Results:**

At baseline, there was no detectable difference in copy number (copies/μL) of methylated (*p* = 0.74) or unmethylated INS cfDNA (*p* = 0.34) between PCOS and Control groups. At follow up, neither methylated (*p* = 0.50) nor unmethylated INScfDNA levels (*p* = 0.48) differed significantly between these groups. Likewise, when pooling the groups, there was no difference between baseline and follow up, in terms of copies of methylated or unmethylated INS cfDNA (*p* = 0.38 and *p* = 0.52, respectively). There were no significant correlations between counts of unmethylated or methylated cfDNA and the clinical measurements of β-cell function and pre-diabetes.

**Conclusion:**

The circulating level of unmethylated and methylated INScfDNA is similar between PCOS and Controls and cannot be used to predict islet β-cell loss and progression to Type 2 diabetes in a 6-year follow-up.

**Trial registration:**

The Danish Data Protection Agency (REG-31-2016. Approval: 01-12-2015) and by the Danish Scientific Ethical committee of Region Zealand (Journal no. SJ-525. Approval: 13-06-2016), Clinicaltrials.gov, (NCT03142633, registered 1. March, 2017, Retrospectively registered).

**Electronic supplementary material:**

The online version of this article (10.1186/s12958-019-0478-7) contains supplementary material, which is available to authorized users.

## Introduction

Polycystic ovary syndrome (PCOS) is the most common endocrine disorder of reproductive age women [1]. It is characterized by hyperandrogenism, polycystic ovary morphology, and anovulation, but also by metabolic disturbances; increased risk of hypertension, dyslipidemia, insulin resistance, impaired glucose tolerance (IGT) and an increased lifetime risk of Type 2 diabetes mellitus (T2D) [1, 2]. Preventive advice and monitoring for these future complications are important. However, it is controversial how to monitor women with PCOS according to the risk of T2D and the guidelines present with some inconsistencies. The latest recommendation is that measurements of HbA_1C_, fasting glucose or oral glucose tolerance test (OGTT) should be repeated with 1 to 3 years interval, according to additional risk factors and an OGTT should be offered when planning pregnancy or seeking fertility treatment [1]. There is a general consensus that the risk of diabetes increases, corresponding to age and increasing BMI [2, 3], but to date, no definitive marker of these metabolic disturbances and T2D has been identified in women with PCOS. Further, the heterogeneous nature of PCOS with differences in metabolic risk profile [4, 5] clarifies the need for new biomarkers.

The abundance of insulin cfDNA that is unmethylated at the − 69 position of the Insulin gene has been suggested as a biomarker of β-cell death, as this site is unmethylated in insulin-producing islet β-cells but methylated in non-islet cells [6–8]. Methylation of a certain genomic region is generally regarded as a deactivation mechanism, and is conducted by DNA methyltransferases (DMT) that catalyze the addition of a methyl group from S-adenosyl-methionine to the carbon in 5′ of cytosine residues in cytosine-phosphate-guanine (CpG) dinucleotides [9]. The active, unmethylated form of the insulin gene from β-cells is presumed to be released into the circulation from dying/dead islet β-cells [10]. Therefore, the amount of circulating unmethylated cfDNA would be a reflection of β-cell death, while methylated cfDNA is suggested to be a marker of cellular stress and death of cells/tissues that do not transcribe insulin [11]. An intense search for methods to quantify methylated and unmethylated insulin cfDNA is ongoing, as it would enable us to diagnose Type 1 Diabetes (T1D) and β-cell destruction before the loss of a significant number of insulin-producing cells. A crucial step in this process has been to identify CpG-sites within the insulin gene that are unique to islet β-cells. There are different approaches to this issue and no consensus of which sites and methods to apply [10, 12, 13].

Though insulin resistance, hyperglycemia and impaired glucose tolerance play a central role in T2D, these alone are not sufficient to lead to T2D without a β-cell defect [14]. In patients with early stages of T2D, β-cells secrete excessive insulin and expand their mass to compensate for the increased metabolic load and obesity-associated insulin resistance. After a period, the β-cell function then deteriorates along with loss of β-cell mass and apoptosis [15, 16]. It is suggested that epigenetic modulations could contribute to these alterations in β-cell function [17]. Currently, only a few groups have demonstrated the methylation pattern of the insulin gene in patients with T2D, and most of them only within the pancreatic tissue of donors, mouse models or pancreatic cell lines [6, 7, 18, 19]. Although one study demonstrated that at-risk subjects who progressed to T1D had higher levels of unmethylated insulin-coding cell-free DNA (INScfDNA) compared with healthy controls [20], there is no knowledge of whether an increased amount of unmethylated circulating INScfDNA could serve as a predictor of T2D, as there are no longitudinal studies of cohorts with an increased risk of T2D.

Women with PCOS are known to have a higher risk of IGT and IFG progressing to T2D [2]. Some studies indicate that the IGT is accompanied by a β-cell dysfunction in PCOS women especially in those with a family history of T2D [21] and might even occur prior to changes in stimulated insulin or glucose levels [20]. Since islet β-cell death is a common denominator to T1D as well as T2D, we aimed to assess if islet β-cell death, measured as circulating unmethylated INScfDNA could serve as a biomarker for risk stratification in PCOS women who progress to prediabetes or T2D.

Droplet digital (dd) PCR was used to measure the copy number of INScfDNA in women with PCOS compared with controls at baseline and at 6 years of follow-up using the method described by Fisher et al. [13]. We also investigated whether the abundance of circulating INScfDNA correlated with markers of insulin resistance and T2D in women with PCOS.

## Methods

### Study cohort

This study was a longitudinal cohort study consisting of 40 women between age 23 and 38, diagnosed with PCOS according to the Rotterdam 2003 criteria [22] and 8 healthy age-match controls, who were recruited at the Fertility Clinic at Holbæk Hospital, Denmark, as a part of the PICOLO cohort [23]. They were examined prior to fertility treatment (Baseline (BL)). The participants were invited for a physical re-examination 6 years later, where an OGTT, transvaginal ultrasound, and blood sampling were performed (Follow-up (FU)). Inclusion criteria: Former participants of the PICOLO study. Exclusion criteria: Oral Contraceptives (OCP) within 8 weeks from examination, endocrinological disease (i.e. type 1 and type 2 diabetes, thyroid dysfunction), severe endometriosis, premature ovarian insufficiency, breastfeeding women and pregnancy.

The study was designed and performed according to the Declaration of Helsinki II and approved by the Danish Data Protection Agency (REG-31-2016) and by the Danish Scientific Ethical committee of region Zealand (Journal no. SJ-525). All subjects gave written consent prior to inclusion.

### Anthropometric measurements

Anthropometric and biochemical measurements obtained in this study population have been described previously [23]. Clinical hyperandrogenism was evaluated with Ferriman Gallway score. Blood Pressure was considered elevated If > 140/90 mmHg [24]. Being overweight was assessed by BMI > 25 kg/m^2^ and abdominal fat by waist-hip circumference (Considered Elevated if waist-hip circumference > 0.85).

### Oral glucose tolerance test (OGTT)

The OGTT was performed after an overnight fast (at least 8 h). We collected venous blood samples and measured glucose, plasma insulin and C-peptide at − 5, 0, 30 and 120 min after a 75 g glucose load. Impaired fasting glucose (IFG) was defined as fasting plasma glucose (FPG) > 5.6 mmol/L and < 6.9 mmol/L and IGT as 2-h plasma glucose between 7.8 mmol/L and 11.0 mmol/L [24].

### Blood sample preparation

Venous blood for digital droplet PCR (ddPCR) was collected in an EDTA tube and kept on ice until centrifugation. Plasma and cellular fractions were separated by centrifugation at 1800Xg for 20 min. Plasma was carefully removed leaving 0.5 mL in order to avoid disturbance of the interface. The EDTA plasma was stored at -80 °C until analysis.

### DNA extraction from plasma

DNA extraction was performed with QIAmp DNA Blood Mini Kit (Qiagen) with 10 μg poly-A DNA as a carrier. All samples were thawed on ice and buffers were prepared according to the manufacturer’s recommendations. We used 50 μL EDTA plasma from a first time thaw aliquot for DNA extraction. All samples then underwent bisulfite conversion to convert unmethylated cytosine into uracil using EZ DNA Methylation-Lightning Kit (Zymo Research). Cell-free DNA concentrations were measured with Qubit™ 4 Fluorometer (Thermofisher Scientific).

*Methylated and unmethylated INScfDNA quantification using digital droplet PCR (ddPCR):*

A MasterMix was prepared using custom SNP TaqMan primer/probes [6], that detected methylation or unmethylation at the CpG site located 69 bp upstream of the transcription start site (TSS). Primer/probe sequences are listed in Additional file 1. Droplets were generated using an automated droplet generator and copies of INScfDNA quantitated using a dual fluorescent probe-based multiplex assay. The selected probe [13] distinguishes DNA that is differentially methylated at nt − 69 of the human insulin gene. Plasmids for INScfDNA (unmethylated, methylated or combinations of these) were used on each assay plate as positive controls. Inter- and intra-assay CVs were less than 5% and were satisfactory. The total INScfDNA (copies/μl) represents the sum of the copies of unmethylated as well as methylated INScfDNA from each plasma sample.

We added 22.5 μL MasterMix, followed by 2.5 μL of bisulfite converted (bc) DNA into each well of a 96-well Eppendorf plate. The PCR reaction-oil droplets were generated in the Auto Droplet Generator (BioRad) followed by sealing of the plate and thermal cycling under following conditions: 95 °C for 10 mins, 40 cycles (94 °C for 30 s, 57.5 °C for 1 min), 98 °C for 10 mins, 12 °C hold. The droplets were analyzed by a QX200 Droplet Reader and QuantaSoft Software (BioRad), from which a concentration (copies/μL) of methylated and unmethylated cfDNA was obtained.

### Statistics

Statistical analyses were executed in IBM Statistical Packages for Social Sciences (SPSS, version 25) and GraphPad Prism (Version 7.04, GraphPad Inc., La Jolla, CA, U.S.A.) All clinical data are presented as means (standard deviation (SD)) or medians (interquartile range (IQR)). All data were tested with Shapiro-Wilks test for normal distribution. For statistical analysis, non-normal distributed data were log transformed and compared with students t-test for paired or non-related samples. INScfDNA was not normal distributed after log transformation. Therefore, INScfDNA is presented as copies/μL and unmethylated/methylated ratio. The differences between groups were assessed using Kruskal-Wallis test and Wilcoxon Signed rank test for repeated samples. Correlations were assessed with Spearman’s correlation for nonparametric data. A *P*-value of less than 0.05 was considered signficant.

## Results

### Demographics of the study population

Basic anthropometric and biochemical characteristics are shown in Table 1. Mean follow-up time was 5.8 years (SD 0.8) (median: 6.1(min 4.0, max 7.1)). Of the 48 women, 33 PCOS (82.5%) and 6 controls (75.0%) gave birth to at least one child between the BL examination and FU. One control (2.6%) and one PCOS (2.6%) were diagnosed with gestational diabetes and two (5.1%) (both PCOS) with preeclampsia during pregnancy.

**Table 1:** Characteristics of the participants

	Baseline			Follow-up			Baseline vs. Follow-up	
	Control *n* = 8	PCOS *n* = 40	p	Control *n* = 8	PCOS *n* = 40	p	Controls p	PCOS p
Age (years)	30.0 (5.2)	29.1 (4.1)	NS	35.6 (6.0)	34.7 (4.2)	NS	–	–
Weight (kg)	72.3 (12.4)	77.6 (20.1)	NS	79.2 (12.8)	78.5 (17.8)	NS	0.03	NS
Height (cm)	169.8 (3.8)	168.5 (7.1)	NS	171.3 (4.3)	168.5 (6.9)	NS	NS	NS
BMI (kg/m^2^)	24.83 (4.2)	26.7 (5.2)	NS	27.0 (4.3)	27.7 (6.1)	NS	0.03	0.013
Waist (cm)	83.4 (8.5)	89.5 (12.8)	NS	90.8 (11.0)	91.7 (15.0)	NS	0.02	0.018
Hip (cm)	106.6 (7.8)	107.8 (9.1)	NS	108.0 (8.0)	109.2 (11.5)	NS	NS	0.018
Waist-Hip Ratio	0.8 (0.1)	0.8 (0.1)	NS	0.8 (0.6)	0.8 (0.1)	NS	0.06	NS
Systolic Blood Pressure (mmHg)	123.2 (17.2)	122.5 (11.0)	NS	113.6 (18.1)	121.0 (12.9)	NS	NS	NS
Diastolic Blood Pressure (mmHg)	76.6 (13.5)	77.0 (10.8)	NS	71.5 (17.1)	72.8 (11.2)	NS	NS	0.04
Ferriman Gallwey Score	2.5 (1.5–4.5)	5.0 (3.0–10.0)	0.019	1.5 (0–5.5)	6.0 (3.0–9.0)	NS	NS	NS
Total testosterone (nmol/L)	0.9 (0.7–1.6)	1.9 (1.4–2.5)	0.001	0.7 (0.4–0.9)	1.4 (1.0–2.0)	0.02	0.039	< 0.001
Free testosterone (nmol/L)	0.019 (0.012–0.023)	0.032 (0.019–0.050)	0.016	0.012 (0.007–0.014)	0.023 (0.014–0.036)	NS	NS	0.008
DHEAS (Umol/L)	5181 (1962)	5676 (2764)	NS	4437 (2550)	4857 (2190)	NS	NS	0.017
Androstenedione (nmol/L)	4.4 (3.0–5.7)	7.1 (4.7–9.0)	0.008	2.8 (1.8–3.4)	5.8 (4.0–8.5)	< 0.001	0.001	0.048
SHBG (nmol/L)	62.5 (45.0–73.5)	59.5 (40.0–83.0)	NS	49.0 (30.0–72.5)	46.0 (36.5–83.0)	NS	NS	0.011
LH/FSH ratio	0.7 (0.5–1.2)	1.7 (1.2–2.3)	< 0.001	0.8 (0.8–1.1)	1.6 (1.1–2.2)	0.02	NS	NS
Fasting s-glucose (mmol/L)	5.3 (0.8)	5.1 (0.4)	NS	4.8 (0.7)	4.9 (0.5)	NS	NS	0.029
s-insulin (pmol/L)	58.8 (40.8–84.1)	45.7 (33.0–104.1)	NS	56.7 (38.6–110.9)	60.1 (37.7–108.9)	NS	NS	0.014
s-C-peptide (nmol/L)	0.6 (0.6–0.7)	0.6 (0.4–0.8)	NS	0.7 (0.6–0.7)	0.7 (0.5–1.0)	NS	NS	< 0.001
HOMA IR (mU*mmol/L^2^)	13.9 (10.1–18.9)	10.3 (7.4–22.7)	NS	11.0 (8.2–27.8)	12.9 (7.6–24.8)	NS	NS	0.056
Total cholesterol (mmol/L)	4.7 (0.7)	4.6 (0.8)	NS	4.3 (0.8)	4.5 (0.8)	NS	NS	NS
LDL cholesterol (mmol/L)	2.9 (0.7)	2.6 (0.7)	NS	2.5 (0.7)	2.6 (0.8)	NS	NS	NS
HDL cholesterol (mmol/L)	1.4 (0.2)	1.60 (0.5)	NS	1.4 (0.3)	1.6 (1.2)	NS	NS	NS
Triglyceride (mmol/L)	0.70 (0.7–0.8)	0.7 (0.6–1.2)	NS	0.9 (0.9–1.2)	0.8 (0.7–1.6)	NS	NS	0.001

Legend: Baseline characteristics of the study participants. Data are presented as mean ± SD or as medians (interquartile range) if not normally distributed. Values were considered significantly different at *P* < 0.05. Abbreviations: *HOMA-IR* homeostatic model assessment of insulin resistance, *HDL* high-density lipoprotein, *LDL* low-density lipoprotein, *LH* Luteinizing Hormone, *FSH* Follicle Stimulating Hormone, *DHEAS* Dehydroepiandrosterone, *SHBG* Sex Hormone-Binding Globulin, *NS* not significant

There were no significant differences in weight, BMI, waist-hip ratio, systolic and diastolic blood pressure between the 2 groups, neither at BL nor at FU. Comparing BL and FU, both groups had increased their BMI and waist measurement, whereas only the controls had significantly increased weight and waist-hip-ratio at follow-up. The PCOS group had significantly increased their hip-measurements, but decreased their diastolic blood pressure.

As expected, the PCOS group had increased levels of androgens (Total Testosterone (T), Free T and Androstenedione) and the ratio between luteinizing hormone and follicle-stimulating hormone (LH/FSH ratio) at BL and at FU compared with controls, although Free T levels were not significantly different between PCOS and controls at FU. Both groups significantly decreased their levels of Total T and androstenedione during the FU time. The PCOS group had further decreased their levels of Free T, Dihydroepiandrosterone-sulfate (DHEAS) and sex hormone binding globulin (SHBG).

At baseline two controls (4.3%) and three PCOS (6.4%) were prediabetic (defined by IGT or IFG). At follow-up, one control (2.2%) and seven PCOS (14.6%) were prediabetic. None of the participants was diagnosed with T2D at baseline or at follow-up. There were no significant differences in fasting glucose, fasting insulin, fasting c-peptide, homeostatic model assessment of insulin resistance (HOMA-IR) between the controls and PCOS at BL or at FU. However, only the PCOS group displayed significant increases in insulin, c-peptide, HOMA-IR and triglycerides between BL and FU.

### Digital droplet PCR analysis of methylated and unmethylated insulin promotor cfDNA in serum

The number of serum samples without detectable levels of unmethylated INScfDNA was 23 (47.9% (17 PCOS (42.5% of PCOS) and 6 controls (75.0% of controls))) and 21 (43.8% (17 PCOS (42.5% of PCOS) and 4 controls (50% of controls))) at BL and FU, respectively. Nine (18.8% (6 PCOS (15.0% of PCOS) and 3 controls (37.5% of controls)) and seven (14.6% (6 PCOS (15.5% of PCOS) and 1 control (12.5% of controls)) samples from each visit, respectively, had no or undetectable levels of methylated INScfDNA.

**Fig. 1:**
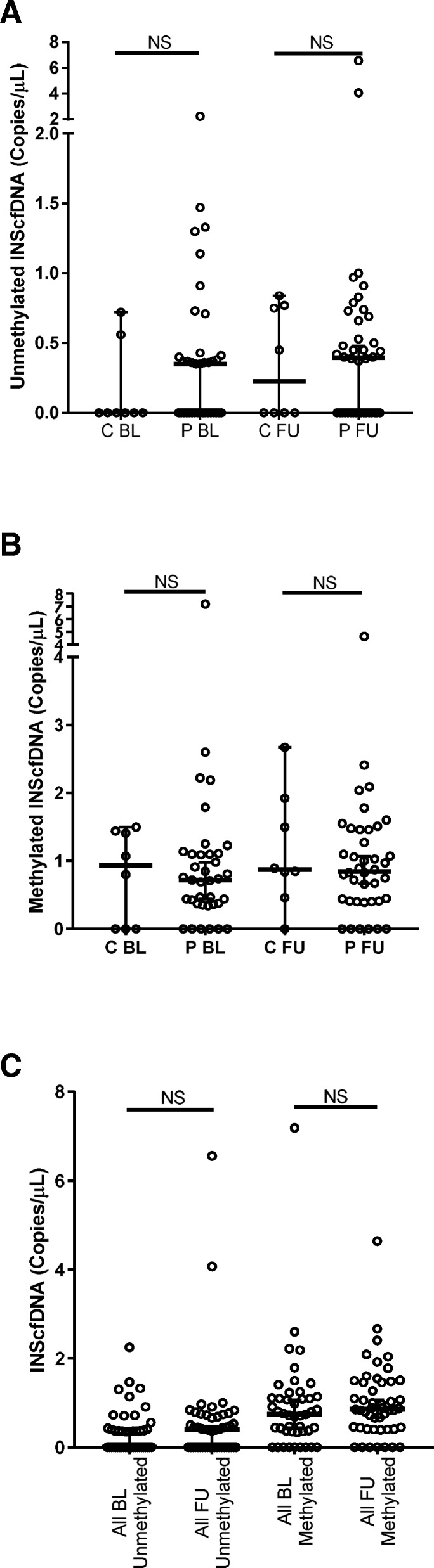
Panel **a**: Methylated INScfDNA. Panel **b**: Unmethylated INScfDNA. Panel **c**: Baseline vs. Follow-up. Number of copies/mL of respectively unmethylated (**a**) and methylated (**b**) INScfDNA. PCOS and control group compared at baseline (BL) and follow-up (FU). BL and FU for all samples expressed by copies/uL, are compared in panel **c**. Results are expressed as medians (95%CI). NS: Not significant

When comparing controls and PCOS for counts of unmethylated INScfDNA (copies/μL), there was no significant difference at baseline (*p* = 0.27) or at follow-up (*p* = 0.99) (Fig. 1a). Similarly, when we compared the counts of methylated insulin promotor cfDNA, there was no significant difference between the PCOS and control groups at baseline (*p* = 0.94) and follow up (*p* = 0.50) (Fig. 1b).

**Fig. 2:**
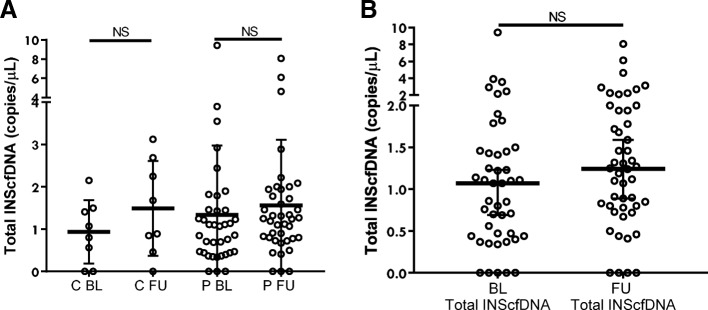
Panel **a**: Total INScfDNA. Panel **b**: Total INScfDNA. Total amount of INScfDNA (umethylated + methylated copies/uL). PCOS and controls are compared in panel **a** and BL vs FU in panel **b**. Results are expressed as medians (95%CI). NS: Not significant

Because there was no difference in levels of INScfDNA between PCOS and controls, we pooled the data from each group at BL and at FU to increase power. However, after pooling these data, there was no difference in unmethylated or methylated INScfDNA, when comparing baseline and follow-up values (*p* = 0.43 and *p* = 0.38) (Fig. 1c). Further, there were no differences in total amount of total INScfDNA (unmethylated + methylated INScfDNA) between the groups neither at baseline nor at follow-up (BL: *p* = 0.74 and FU: *p* = 0.79) (Fig. 2a). The total amount of INScfDNA showed no differences for all participants between baseline and follow-up (*p* = 0.25) (Fig. 2b).

**Fig. 3:**
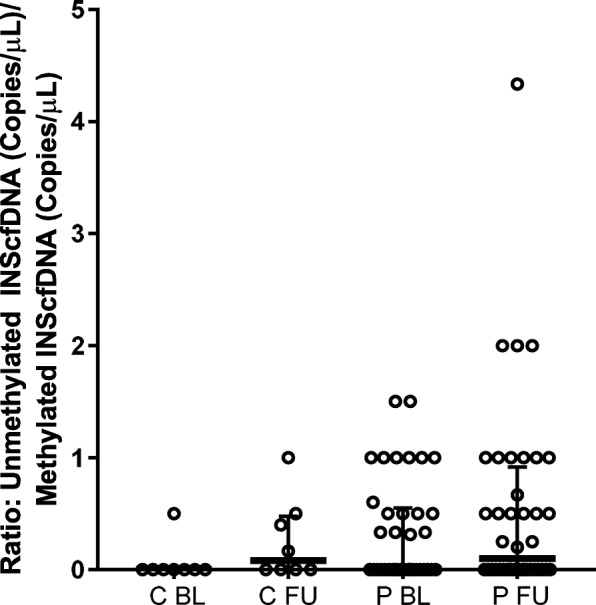
Ratio Unmethylated/methylated INS cfDNA. Ratio between unmethylated and methylated INS cfDNA (copies/μL/copies/μL). Results are expressed as medians (95%CI)

Figure 3 shows the ratio between unmethylated and methylated insulin cfDNA. When comparing ratios for the PCOS versus the control group at BL and FU, there were no significant differences (*p* = 0.18 and *p* = 0.48). Likewise, no differences were detected during the 6 yr. FU for each group (controls *p* = 0.13 and PCOS *p* = 0.52).

### Subanalysis of unmethylated and methylated insulin cfDNA in prediabetic and glucose tolerant participants

Since the number of baseline (*n* = 2) and follow-up (*n* = 1) controls with prediabetes (defined by IFG or IGT) were very low, it was not possible to conduct analysis on prediabetic versus glucose-tolerant participants individually in the groups of PCOS and controls. Comparing methylated INScfDNA for all prediabetic participants (both controls and PCOS) with all glucose tolerant participants, no difference were seen neither at baseline (*p* = 0.72) nor at follow-up (*p* = 0.07) (Additional file 2). A similar nonsignificant result were seen for unmethylated INScfDNA values at baseline and at follow-up (*p* = 0.27 and *p* = 0.11) (Additional file 2)). To analyze the predictive capability of methylated or unmethylated INScfDNA, we compared the baseline levels of INScfDNA for the participants who were diagnosed with prediabetes at FU with those who were glucose tolerant at follow-up. This analysis showed no significant differences in unmethylated INScfDNA (*p* = 0.53) or methylated INScfDNA (p = 0.07). A binary logistic regression model including BMI revealed no further significant predictive utility of unmethylated INScfDNA (*p* = 0.74) or methylated INScfDNA (*p* = 0.65).

### Association of unmethylated and methylated insulin cfDNA and clinical markers of metabolic syndrome or PCOS

No strong statistically significant correlations were identified between unmethylated or methylated insulin cfDNA and weight, BMI, waist-hip ratio, blood pressure, lipids (total cholesterol, HDL- or LDL-cholesterol and triglyceride), insulin, c-peptide, glucose, HOMA-IR or androgen status (FG-score, free testosterone, total testosterone, androstenedione, SHBG and DHEAS) (Additional file 3)), neither at baseline nor at follow-up.

## Discussion

Islet β-cell death is known to be a common denominator in progression to type 1 or type 2 diabetes. Β-cell death in T2D is well known to be associated with exogenous insulin requirement. However, there are several lines of evidence [25] indicating that progression to Type 2 diabetes itself may result following loss of functional islet β-cells. Thus, our study aimed to measure biomarkers of islet β-cell death (i.e INS cfDNA) in PCOS/Control women who would develop T2D in future. At the moment, there is a consensus that elevated levels of unmethylated INScfDNA correlate with β-cell death and T1D in mice and humans [8, 10, 12], whereas methylated cfDNA is suggested as a marker of inflammation or sepsis [11]. With this study, we investigated whether this relatively new method could be used for detecting early signs of β-cell death and T2D in women with PCOS. Our results could not confirm this hypothesis, as we did not find any differences in methylated or unmethylated INScfDNA when we compared PCOS women with healthy controls. Likewise, we could not detect any increase in methylated or unmethylated INScfDNA over a period, even though the PCOS group presented with a more metabolically challenged profile at FU. Neither did we find a correlation between markers of insulin resistance and metabolic syndrome and insulin promotor DNA methylation status.

There are several issues that could contribute to these negative findings. First: the insulin gene and its promotor contain several methylation sites, but not all of them display a unique methylation pattern in β-cells compared with other tissues [26]. The tissue specificity of these methylation sites is essential for their usefulness as biomarkers of β-cell death. The evidence though is still sparse on this and there is no consensus of which sites that are the best determinants of islet β-cell stress or death and of future progression to T1D. Previous studies have also used the CpG site (− 69) upstream the TSS of the insulin gene, and reported increased levels of both unmethylated and methylated insulin cfDNA in patients with T1D, but not in patients with T2D [6]. Another study [27] primarily tested downstream CpG sites (located at + 255, + 273, + 303, + 329, + 364, + 370, + 396 and + 399) within the coding region of the insulin gene and confirmed increased levels of unmethylated cfDNA in patients with T1D as well as subjects with increased risk of T1D [28]. A third group identified a cluster of six other CpG sites, within the insulin promoter, with Illumina 450 K methylation arrays and next-generation sequencing, that was able to distinguish between healthy controls and patients with T1D [29].

Secondly, little research into INScfDNA has been done in patients with T2D, and none on women with PCOS. Likewise, the existing studies report results with some discrepancy. Studies indicate that the DNA methylation is not stable and not all CpG sites are consistently methylated during periods of cellular stress, which could be an explanation to the inconsistency in findings [7]. Further, it is most likely that the DNA methylation during T2D occurs at multiple loci with small effect sizes that contribute to an increased risk for disease [30]. Heterogeneity in the methylation sites affected would decrease the sensitivity of a cfDNA biomarker.

Fisher et al. [6] used the CpG site (− 69) and found that neither methylated nor unmethylated cfDNA levels were increased in patients with T2D compared with controls. Another group investigated the DNA methylation in 25 CpG sites within the insulin promoter and gene in pancreatic islets from patients with T2D and controls [7]. They reported that four sites (located at − 234, − 180, − 102 and + 63) showed increased methylation in pancreatic β-cells from patients with T2D compared with controls. Furthermore, they showed that the percentage of DNA-methylation was positively correlated to HbA_1C_, but negatively correlated with insulin mRNA expression. [7] These findings indicate that increased DNA methylation of the insulin promotor in the pancreas could be a contributor to the downregulation of insulin expression in T2D as a pathological response to hyperglycemia. This is in line with the findings of Kenna et al. [31] who investigated the methylation pattern in women with gestational diabetes compared with pregnant women without gestational diabetes. They suggested that a decreased fraction of unmethylated insulin promotor DNA is a reflection of a decreased β-cell turnover and the decreased turnover could be a mechanism to compensate for the need for higher insulin levels.

Third, PCOS is a heterogeneous condition – there is variation within the group mainly explained by the syndrome definition of PCOS [1, 32], but also variation over time caused by e.g. pregnancy or interventions in lifestyle [33–35]. Some studies show that the different PCOS phenotypes differs in cardiometabolic risk, with the highest risk in hyperandrogenic and anovulatory phenotypes [4, 5]. However, we did not detect any differences in unmethylated or methylated INScfDNA neither at baseline nor at follow-up in a subanalysis of PCOS phenotypes based on Rotterdam criteria (data not shown). Further, the cohort was recruited when referred to assisted reproductive treatment (ART), and as a standard procedure, all PCOS women with a BMI > 25 are asked to initiate lifestyle interventions. Looking at weight only, the control group increased theirs during FU, while the PCOS group did not. The steady weight in the PCOS group could be interpreted as an alteration in general lifestyle. The fact that our cohort is recruited prior to ART, could also have caused selection bias, as those women with need of ART possibly are challenged with a severe PCOS phenotype. Moreover, our participants were relatively young at the follow-up time point in terms of developing insulin resistance, prediabetes or T2D. The effect on INScfDNA methylation pattern could have been more pronounced if the participants were older or if the follow-up time had been longer. Another explanation to the negative results is a possible effect of PCOS status on liver function or the use of metformin in the PCOS women. PCOS women are known to have an increased risk of developing non-alcoholic fatty liver disease (NAFLD) [36], and although there is no studies on NAFLD and INScfCNA counts, one study have indicated that the presence of autoimmune hepatitis has a decreasing effect on levels of methylated and unmethylated INScfDNA compared with controls [6]. This could cause an underestimation of the differences between the groups and thereby our results. The use of metformin could also be a confounder in our study. There are no studies of the effect of metformin on INScfDNA counts, but insulin treatment in newly diagnosed T1D is shown to reduce both methylated and unmethylated INScfDNA significantly [6]. If the effect of metformin is similar, use of metformin in the PCOS group could also cause an underestimation of the differences between the groups. Unfortunately, we have no reliable records of the metformin use in our cohort between baseline and follow-up. Finally, one could argue that our sample size, and especially the control group, is too small. This study was designed with an assumption of that, in a PCOS cohort aged 30–40 and BMI > 25, the prevalence of prediabetes (IGT or IFG) would be approximately 40–45% [37] at follow up. The prevalence in our cohort did not fulfill this assumption, as only 17.5% of our PCOS cohort was prediabetic, causing very low power. If the prevalence had been as expected, the power would had been acceptable. The lower number of participating controls resulted due to a recruitment issue, which might cause an underestimation of the differences between PCOS and controls.

We were not able to detect any differences in unmethylated or methylated INScfDNA at any time in this study. However looking at samples with undetectable levels INScfDNA, there are a relatively larger number of samples without detectable levels of unmethylated INScfDNA, than of methylated INScfDNA. This advocates the theory of PCOS as an inflammatory condition with increased cell-turnover in general, as suggested by others [38, 39], even though we did not detect significant differences between controls and PCOS.

## Conclusion

The cell-free DNA methylation pattern of the insulin gene promoter has been suggested as a marker of pancreatic islet β-cell destruction, and thereby as an early marker of diabetes. Since islet β-cell death is a common denominator in T1D as well as T2D, we explored if circulating insulin cell-free DNA can determine differences in BL and 6 year follow-up samples in a cohort of women with PCOS or without. We were not able to detect any differences in the levels of INScfDNA between PCOS and controls at BL as well as over a period of 6 years. Although these negative findings could be a result of the heterogeneous nature of PCOS or by the relatively short follow-up time, the publication of negative findings is an important contribution to scientific understanding of a topic, avoiding publication bias by preferentially publishing only papers with positive results increasing the risk that an incorrect prevailing view can persist. Further studies in larger and longer longitudinal cohorts are warranted.

## Additional files

Additional file 1: Primer and probe sequences. Assay ID: AH21BH1, HINSMethyl (DOCX 11 kb)

Additional file 2: Panel A: Methylated INScfDNA Panel B: Unmethylated INScfDNA. Legend: Number of copies/mL of respectively unmethylated (A) and methylated (B) INScfDNA. Glucose tolerant (GT) and prediabetic (PD) participants compared at baseline (BL) and follow-up (FU). (DOCX 43 kb)

Additional file 3: Correlations between clinical measurements and unmethylated and methylated INScfDNA at baseline and at follow-up. (XLSX 14 kb)
