# Supplementary material for: Levels of circulating insulin cell-free DNA in women with polycystic ovary syndrome – a longitudinal cohort study
Source: Reprod Biol Endocrinol. 2019 Apr 5;17:34. doi: 10.1186/s12958-019-0478-7 (PMC6451227; doi:10.1186/s12958-019-0478-7)
Supplement: Supplementary file 1 — Primer and probe sequences. Assay ID: AH21BH1, HINSMethyl (DOCX 11 kb) [file 12958_2019_478_MOESM1_ESM.docx]

|  | Sequence |
| --- | --- |
| Forward Primer | 5’-GGAAATTGTAGTTTTAGTTTTTAGTTATTTGT-3’ |
| Reverse Primer | 5’-AAAACCCATCTCCCCTACCTATCA-3’ |
| Reporter 1 (VIC) | 5’-ACCCCTACC**G**CCTAAC-3’ |
| Reporter 2 (FAM) | 5’-ACCCCTACC**A**CCTAAC-3’ |

Supplementary Material 1
