# Supplementary material for: Levels of circulating insulin cell-free DNA in women with polycystic ovary syndrome – a longitudinal cohort study
Source: Reprod Biol Endocrinol. 2019 Apr 5;17:34. doi: 10.1186/s12958-019-0478-7 (PMC6451227; doi:10.1186/s12958-019-0478-7)
Supplement: Supplementary file 2 [file 12958_2019_478_MOESM2_ESM.docx]

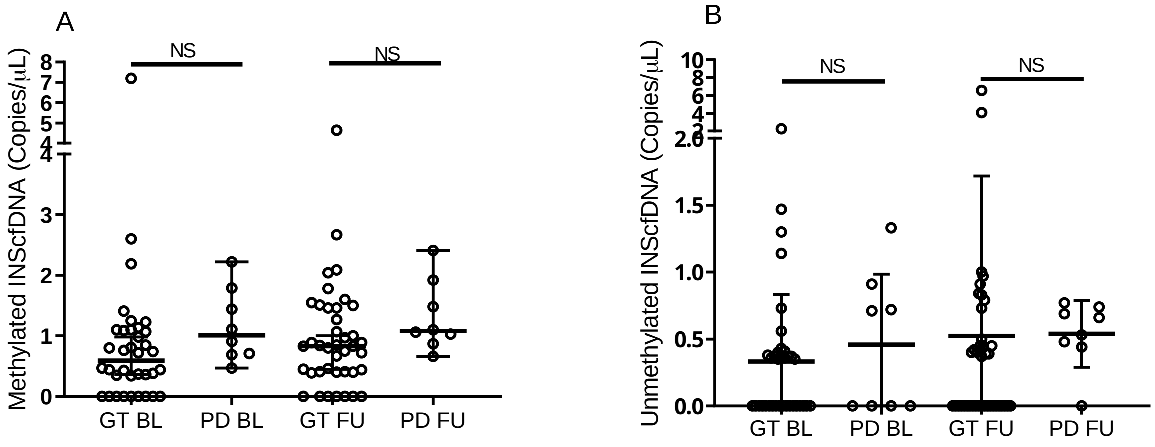

A
NS
NS
Methylated INScfDNA (Copies/μL)
0
1
2
3
4
4
5
6
7
8
GT BL
PD BL
GT FU
PD FU
B
NS
NS
Unmethylated INScfDNA (Copies/μL)
0.0
0.5
1.0
1.5
2.0
2
4
6
8
10
GT BL
PD BL
GT FU
PD FU
